# Salmonella Invasion Is Controlled by Competition among Intestinal Chemical Signals

**DOI:** 10.1128/mbio.00012-23

**Published:** 2023-04-05

**Authors:** Rimi Chowdhury, Paulina D. Pavinski Bitar, Hanora M. Chapman, Craig Altier

**Affiliations:** a Department of Population Medicine and Diagnostic Sciences, Cornell University, Ithaca, New York, USA; University of Washington

**Keywords:** *Salmonella*, enteric pathogens, fatty acids, host-pathogen interactions, infectious disease, pathogenesis mechanisms, virulence regulation

## Abstract

The intestine is a complex, ever-changing environment replete with an array of signaling molecules. To colonize such a complex organ, pathogens have adapted to utilize specific cues from the local environment to intricately regulate the expression of their virulence determinants. Salmonella preferentially colonizes the distal ileum, a niche enriched in the metabolite formic acid. Here, we show that the relatively higher concentration of this metabolite in the distal ileum prevents other signals from repressing Salmonella invasion in that region. We show that imported and unmetabolized formic acid functions as a cytoplasmic signal that competitively binds to HilD, the master transcriptional regulator of Salmonella invasion, thus preventing repressive fatty acids from binding to the protein. This results in an increased lifetime of HilD and subsequent derepression of invasion genes. This study demonstrates an important mechanism by which Salmonella utilizes competition among signals in the gut to its advantage as a pathogen.

## INTRODUCTION

All enteric pathogens share a burden, the burden of virulence. To be successful, pathogens must express their virulence factors, but in most cases expression of these virulence genes is an economic luxury, one difficult to sustain in an extensively resource-limited setting such as the intestine. Therefore, successful enteric pathogens have adapted to strictly control the expression of their virulence genes, and allow their expression only under specific environmental conditions, for maximal pathogenic benefit (1). These bacteria acutely sense their local milieu for specific cues and use them as signals to regulate their virulence processes (2–5). In this way, the enteric pathogen Salmonella scouts for the appropriate location to penetrate the intestinal epithelial layer, a function essential to virulence termed invasion, by sensing specific cues within its local environment. Such spatial cues are then employed as signals that dictate the virulence outcomes of Salmonella in that location (1).

Salmonella employs many environmental cues to modulate the expression of its invasion genes (6–10). These compounds are utilized directly or indirectly to target the master transcription regulators, the AraC-type proteins HilD, HilC, and RtsA. Upon activation, these transcriptional regulators can induce themselves and each other by binding to their DNA operator regions. In this way, they amplify the signal to a threshold that triggers the transcription of the downstream regulator *hilA* and a cascade of invasion genes (6, 11–13). Most of the known environmental signals modulate Salmonella invasion by disrupting the ability of these regulators to bind to DNA and mediate transcription initiation. In the small intestine, Salmonella encounters a high concentration of bile and uses it to prevent the invasion event in that region. Bile functions by disrupting the HilD-DNA complex and mediates rapid degradation of the protein, thus preventing invasion (9). Similarly, the invasion event is avoided in the large intestine by using short- and long-chain fatty acids (S/LCFA), produced by a vast native microbiota (14). Fatty acids such as the propionic and oleic acids function to prevent invasion by disrupting the DNA-binding ability of HilD (6, 7). Another LCFA, *cis*-2-hexadecenoic acid (c2-HDA), a potent repressor of Salmonella invasion found in the murine cecum and colon, directly binds to HilD, HilC, and RtsA with high affinity and disrupts their ability to bind to their target DNA (15, 16). In this way, Salmonella uses environmental compounds as spatial cues to prevent invasion in different regions of the intestine.

Salmonella preferentially invades the distal ileum (17–21). However, the mechanisms by which Salmonella selectively initiates invasion within this distinct and confined region remain unexplored. The murine distal ileum is rich in formic acid, a diffusible signal that can induce invasion gene expression by an unknown mechanism (8). Here, we show that formic acid is selectively enriched in the murine distal ileum compared to cecum and colon. This single-carbon metabolite prevents fatty acids from binding to HilD and thus from disrupting the HilD-DNA complex. Formic acid incubation also significantly increases the lifetime of HilD by preventing its fatty acid-mediated degradation. By this mechanism, formic acid thus significantly increases the invasion gene expression despite the presence of the repressive fatty acids. These results illuminate the molecular mechanism by which Salmonella employs a metabolite selectively enriched in a part of the gut as a derepressor to initiate invasion in that region.

## RESULTS

### Formic acid prevents intestinal fatty acids from repressing Salmonella invasion genes.

**FIG 1:**
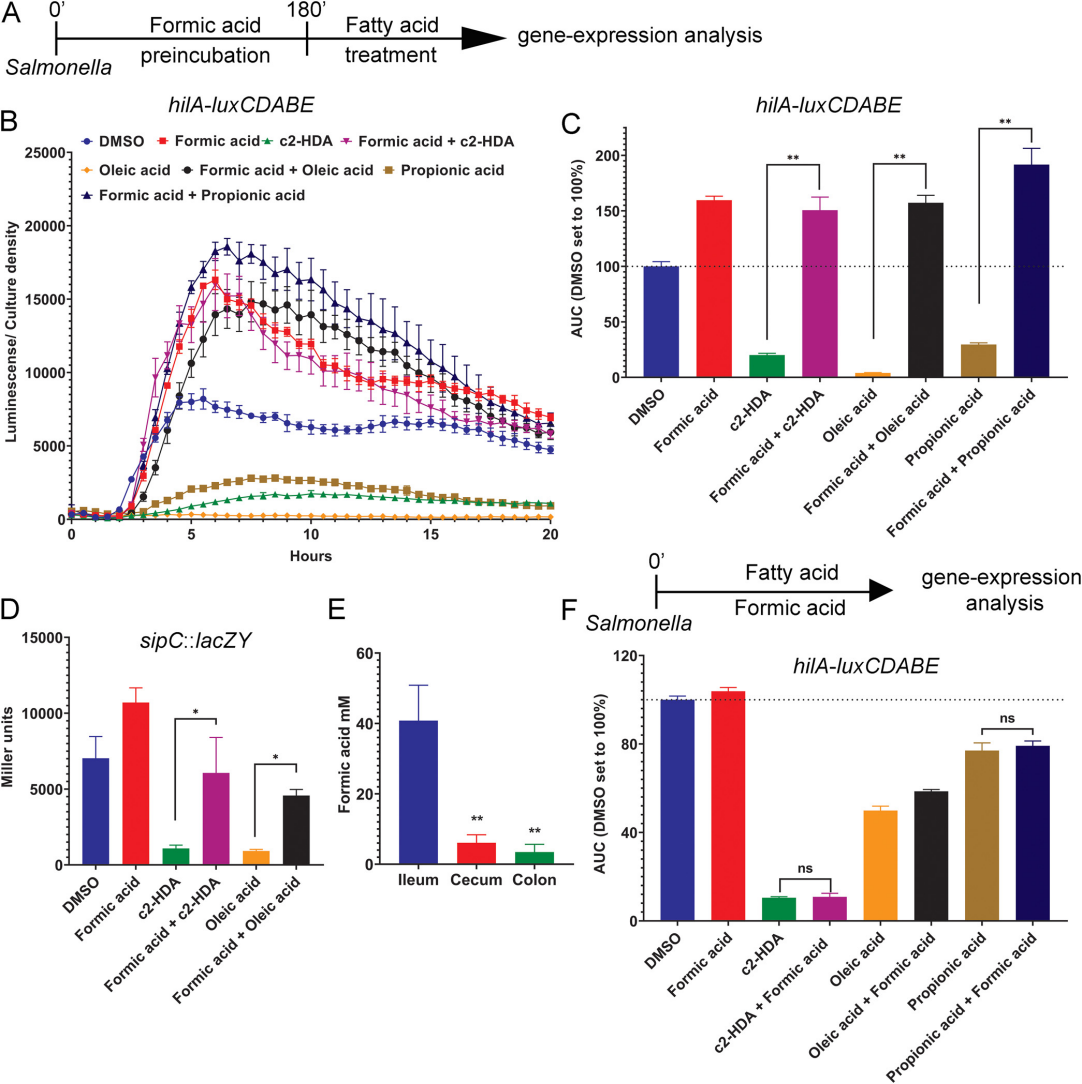
Formic acid prevents intestinal fatty acids from repressing Salmonella invasion genes. (A) Summary of the experimental setup to analyze the expression of invasion genes. (B to C) Expression of the invasion gene *hilA* was determined in Salmonella with different treatments (preincubation with 10 mM formic acid followed by either 500 nM c2-HDA, 40 μM oleic, 10 mM propionic acid, or an equal volume of DMSO) shown using a *hilA-luxCDABE* transcriptional reporter fusion. (B) Luminescence was normalized to bacterial culture density. Data show the mean ± standard deviation (SD) of five replicates. (C) The area under the curve (AUC) for each treatment was calculated. The AUC for DMSO treatment was set to 100%, and others were normalized accordingly. Bars show the normalized AUC ± SD (*n* = 5). Differences between the indicated treatments were calculated with the Mann-Whitney test. **, *P* < 0.01. (D) Expression of the invasion gene *sipC* was evaluated in Salmonella, preincubated with 10 mM formic acid prior to treatment with 10 μM c2-HDA or 40 μM oleic acid, using a *lacZY* transcriptional reporter fusion by β-galactosidase assay. Bars represent the mean ± SD (*n* = 4). Differences between treatments were calculated with the Mann-Whitney test. *, *P* < 0.05. (E) Determination of the formic acid concentration in the intestinal contents of C57BL/6 mice (*n* = 10). Bars represent the mean ± SD. Differences between formic acid concentrations in ileum versus cecum or colon were calculated with the Mann-Whitney test. **, *P* < 0.01. (F) The expression of invasion gene *hilA* was determined in Salmonella treated with 10 μM fatty acids or an equal volume of DMSO and 10 mM formic acid, shown using a *hilA-luxCDABE* transcriptional reporter fusion. Luminescence was normalized to bacterial culture density. The AUC for each treatment was calculated. The AUC for DMSO treatment was set to 100%, and others were normalized accordingly. Bars show the normalized AUC ± SD (*n* = 5). Differences between the indicated treatments were calculated with the Mann-Whitney test. ns, not significant.

Salmonella utilizes specific compounds from its local environments as signals to regulate the expression of its invasion genes (6–10, 15, 22, 23). The environment of the murine distal ileum, the preferred site of Salmonella invasion, is rich in the metabolite formic acid (8). Additionally, formic acid was shown to induce Salmonella invasion gene expression, but the mechanism remains undetermined (8). We hypothesized that formic acid induces invasion gene expression by preventing repressors, such as short- and long-chain fatty acids, from quelling the expression of Salmonella invasion genes. To test this, we compared invasion gene expression in Salmonella preincubated with formic acid followed by treatment with intestinal fatty acids known to repress this function. Using a plasmid-borne *luxCDABE* reporter fusion to the invasion regulator *hilA*, we measured light production over a time course experiment to quantify *hilA* expression. We found that in the absence of formic acid preincubation, *hilA* expression was severely repressed by intestinal fatty acids such as oleic acid and propionic acid but that preincubation with formic acid significantly restored *hilA* expression (by ~40 and ~6.5 fold, respectively) (Fig. 1A to C). *cis*-2 hexadecenoic acid (c2-HDA), a potent invasion repressor found in the murine cecum and the colon, drastically reduced *hilA* expression. However, preincubation with formic acid restored *hilA* expression significantly (~7.5 fold). To determine whether control of this regulator had effects on SPI-1, we quantified the expression of a downstream and terminal invasion gene, *sipC*, using a *sipC*::*lacZY* reporter fusion. Salmonella strains were preincubated with formic acid prior to addition of fatty acids, and *sipC* expression was quantified by β-galactosidase assays. As expected, *sipC* expression was repressed by c2-HDA and oleic acid. In Salmonella that was preincubated with formic acid, however, *sipC* expression was restored significantly (~5.6 fold and ~5 fold, respectively) (Fig. 1D). These results conclusively show that formic acid preincubation prevents intestinal fatty acids from repressing Salmonella invasion-gene expression.

Salmonella invasion is highly restricted in the murine large intestine (21). Therefore, we reasoned that the formic acid concentrations in the cecum and colon may be lower than that of the ileum and thus inadequate to prevent the fatty acids from repressing invasion genes. To test this, we measured the concentration of formic acid in various regions of the murine intestine. The contents of the ileum, cecum, and colon of C57BL/6 mice (*n* = 10) were extracted, and an enzymatic assay was performed to assess formic acid concentrations. We found that the ileum had a significantly higher concentration of formic acid than both the cecum (~7 fold) and the colon (~12 fold) (Fig. 1E). Similar results were obtained in a second mouse strain, BALB/c, and with different age groups (age 6 weeks and age 10 weeks), thus showing that the high localized concentration of formic acid is limited to the murine ileum (see Fig. S1 in the supplemental material). These results may explain, at least in part, how Salmonella invasion is targeted to the ileum, the most distal portion of the small intestine, and reduced in the large intestine.

10.1128/mbio.00012-23.1 FIG S1 Determination of formic acid concentration in the intestinal contents of BALB/C mice (*n* = 5). Bars represent the mean ± SD. Download FIG S1, TIF file, 0.7 MB. Copyright © 2023 Chowdhury et al. 2023 Chowdhury et al. https://creativecommons.org/licenses/by/4.0/ This content is distributed under the terms of the Creative Commons Attribution 4.0 International license.

These results imply that as Salmonella moves from the ileum toward environments with lower concentrations of formic acid, the invasion-rescuing effect of formic acid is lost. As the cecum and the colon have lower formic acid and higher fatty acid concentrations (14), the sequential exposure to these chemical cues may be utilized by Salmonella as a signal to switch from induction to repression of invasion. To test this hypothesis, we analyzed invasion gene expression in Salmonella under various conditions, using a *hilA*::*luxCDABE* reporter fusion. We found that when Salmonella cultures were preincubated with formic acid followed by addition of fatty acids, formic acid significantly rescued the repression by fatty acids. However, when formic acid and fatty acids were added together or when fatty acids were added prior to formic acid, no substantial rescue from repression was observed (Fig. 1F, Fig. S2). These results thus show that the sequence of exposure to different signals plays a crucial role in their effects on Salmonella invasion gene expression and supports a model of spatial and temporal invasion regulation dictated by the changing chemical environment of the gut.

10.1128/mbio.00012-23.2 FIG S2 Temporal regulation of invasion genes. The expression of the invasion gene *hilA* was determined in Salmonella treated with 10 μM fatty acids or an equal volume of DMSO and 10 mM formic acid, shown using a *hilA-luxCDABE* transcriptional reporter fusion. Luminescence was normalized to bacterial culture density. Data show the mean ± SD (*n* = 5). Download FIG S2, TIF file, 2.1 MB. Copyright © 2023 Chowdhury et al. 2023 Chowdhury et al. https://creativecommons.org/licenses/by/4.0/ This content is distributed under the terms of the Creative Commons Attribution 4.0 International license.

### Import of formic acid, but not its metabolism, is required to rescue the expression of invasion genes.

**FIG 2:**
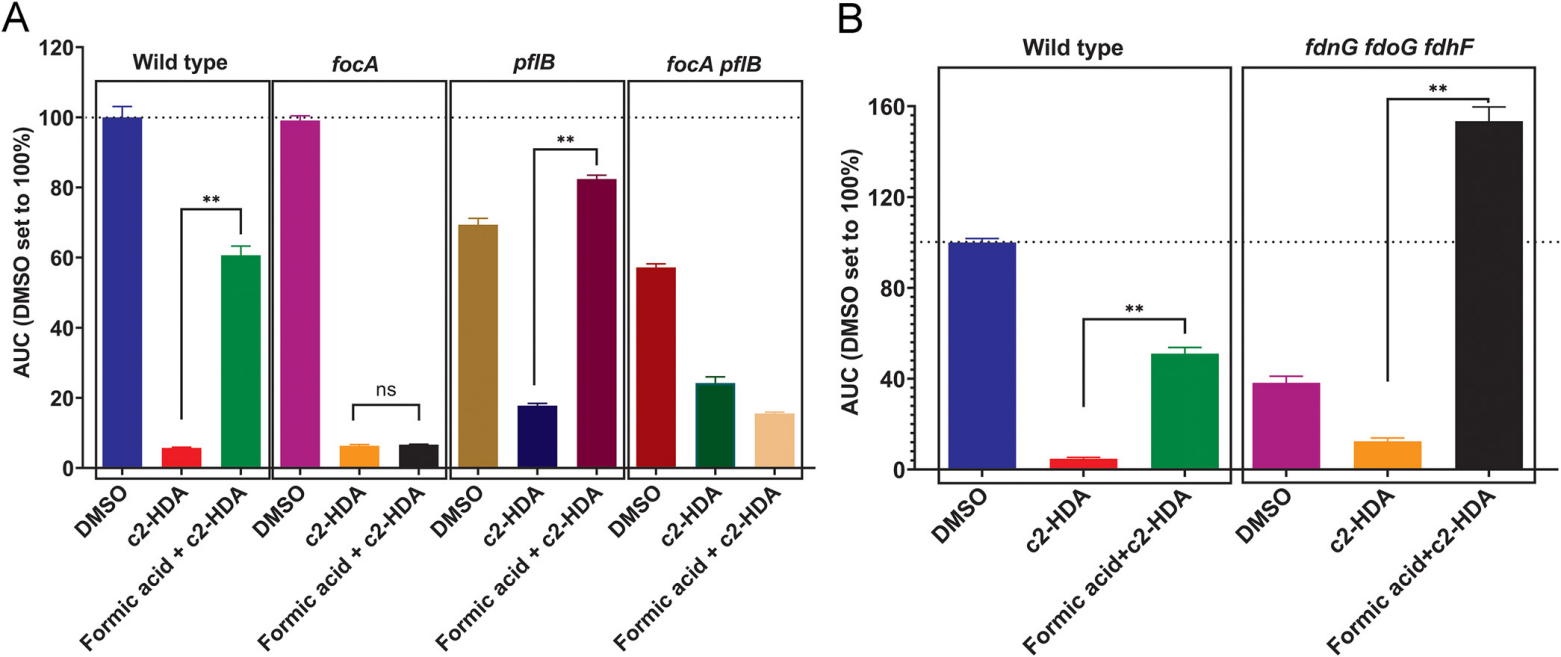
Import of formic acid, but not its metabolism, is required to rescue the expression of invasion genes. (A and B) Expression of the invasion gene *hilA* was determined in Salmonella strains with different treatments (preincubation with 10 mM formic acid before adding 500 nM c2-HDA or an equal volume of DMSO) shown using a *hilA-luxCDABE* transcriptional reporter fusion. Luminescence was normalized to bacterial culture density. The AUC for each treatment was calculated. The AUC for DMSO treatment was set to 100%, and others were normalized accordingly. Bars show the normalized AUC ± SD (*n* = 5). Differences between the indicated treatments were calculated with the Mann-Whitney test. **, *P* < 0.01; ns, not significant.

Salmonella imports exogenous formic acid via the formic acid transport channel protein FocA, encoded by *focA* (24, 25). To determine whether formic acid acts as a cytoplasmic signal to rescue invasion, we tested a *focA* null mutant of Salmonella carrying the previously described *hilA-luxCDABE* reporter plasmid. We found that preventing formic acid import through the deletion of *focA* significantly impaired the ability of formic acid to rescue invasion-gene repression by c2-HDA (Fig. 2A, Fig. S3). Thus, the import of exogenous formic acid is essential to prevent the repression of invasion genes. Salmonella also produces endogenous formic acid by metabolizing pyruvate using the enzyme pyruvate formate lyase, encoded by *pflB*. Deleting *pflB* prevents Salmonella from producing endogenous formic acid (8). To determine if endogenous formic acid plays a role in the rescue of Salmonella invasion genes, we constructed a *hilA-luxCDABE* reporter strain with a *pflB* null mutation and compared the rescue of invasion gene expression in Δ*focA* and Δ*pflB* mutants. As shown in Fig. 2A, both Δ*focA* and Δ*pflB* null mutants were repressed efficiently by c2-HDA. However, preincubation with formic acid rescued invasion gene expression by ~5 fold in the Δ*pflB* mutant, while there was no effect in the Δ*focA* mutant. Additionally, despite formic acid preincubation, the Δ*focA* Δ*pflB* double mutant strain remained repressed by c2-HDA, showing that only the imported formic acid is sufficient to rescue the expression of invasion genes. These results show that the production of endogenous formic acid by Salmonella is insufficient to rescue invasion genes, requiring instead exogenously supplied formic acid to achieve this effect.

10.1128/mbio.00012-23.3 FIG S3 Import of formic acid is required to rescue the expression of invasion genes. Expression of the invasion gene *hilA* was determined in Salmonella strains having null mutations in Δ*focA* or Δ*pflB* or Δ*focA* Δ*pflB* with different treatments (preincubation with 10 mM formic acid before adding 500 nM c2-HDA or an equal volume of DMSO) shown using a *hilA-luxCDABE* transcriptional reporter fusion. Luminescence was normalized to bacterial culture density. Download FIG S3, TIF file, 2.7 MB. Copyright © 2023 Chowdhury et al. 2023 Chowdhury et al. https://creativecommons.org/licenses/by/4.0/ This content is distributed under the terms of the Creative Commons Attribution 4.0 International license.

Salmonella metabolizes formic acid by using either the formate dehydrogenase O or N system, encoded by *fdoGHI* and *fdnGHI*, respectively (8). Formic acid can also be converted to carbon dioxide and water by *fdhF-*encoded formate dehydrogenase (8). It is therefore possible that formic acid itself is the chemical essential to rescue invasion-gene expression or that one or more of its metabolic products serves that role. To test this, we prevented formic acid metabolism by constructing strains with null mutations in *fdnG*, *fdoG*, and *fdhF*, eliminating all known mechanisms of formic acid utilization in Salmonella (8). Using the *hilA-luxCDABE* reporter system, we compared invasion gene repression with fatty acids, with and without formic acid preincubation. We found that formic acid was able to rescue c2-HDA-mediated repression in both the wild-type and the Δ*fdnG* Δ*fdoG* Δ*fdhF* mutant Salmonella (Fig. 2B, Fig. S4), demonstrating that formic acid metabolism is not required for its ability to rescue invasion gene expression. In fact, the inability to metabolize formic acid enhanced *hilA* expression in the Δ*fdnG* Δ*fdoG* Δ*fdhF* mutant Salmonella strain, as would be expected if formic acid were itself the signal. Together, these results show that formic acid supplied by the environment is imported and utilized by Salmonella to prevent its invasion genes from repression by fatty acid signaling molecules present in the intestine.

10.1128/mbio.00012-23.4 FIG S4 Metabolism of formic acid is not required to rescue the expression of invasion genes. Expression of the invasion gene *hilA* was determined in Salmonella strains having null mutations in Δ*fdnG ΔfdoG ΔfdhF* with different treatments (preincubation with 10 mM formic acid before adding 500 nM c2-HDA or an equal volume of DMSO) shown using a *hilA-luxCDABE* transcriptional reporter fusion. Luminescence was normalized to bacterial culture density. Download FIG S4, TIF file, 2.0 MB. Copyright © 2023 Chowdhury et al. 2023 Chowdhury et al. https://creativecommons.org/licenses/by/4.0/ This content is distributed under the terms of the Creative Commons Attribution 4.0 International license.

### Formic acid derepresses invasion gene expression through HilD.

**FIG 3:**
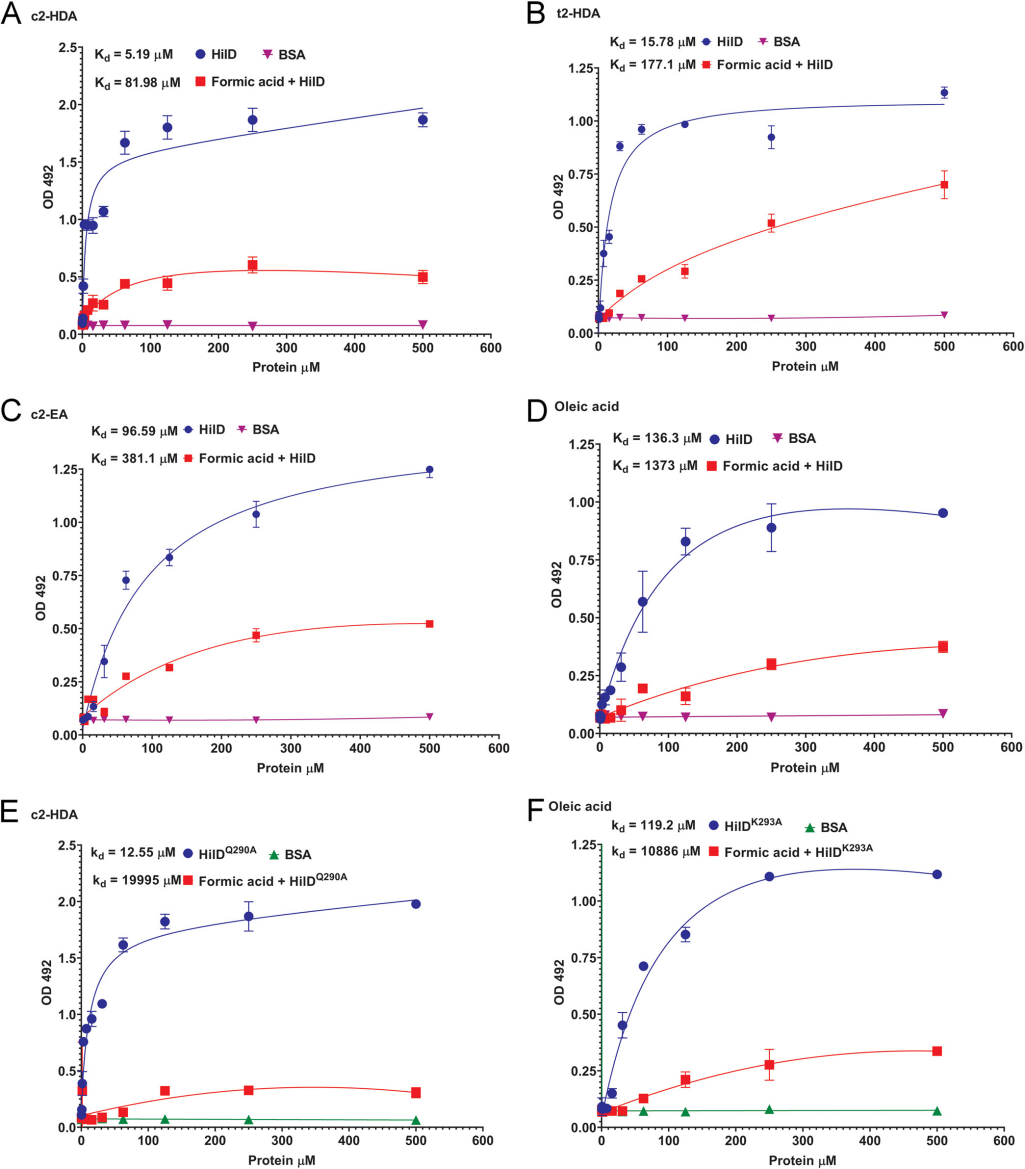
Formic acid derepresses invasion gene expression through HilD. (A to F) The binding of different fatty acids to increasing concentrations of (A to D) wild-type or (E and F) mutant HilD proteins was assessed using ELISA. Nonlinear regression was plotted, and total one-site binding was analyzed to calculate the *K_d_*.

HilD is the master transcriptional regulator of Salmonella invasion genes, with most of the environmental signals that control the expression of Salmonella invasion genes doing so through HilD (7, 9, 15). We have previously shown that intestinal fatty acids directly target HilD to control Salmonella virulence by binding to this essential regulator and preventing its ability to interact with its DNA targets (7, 9, 15). We thus hypothesized that formic acid, consisting of a single carboxylate group, may competitively bind to HilD and thereby prevent repressive fatty acids from interacting with this protein. We initially tested this hypothesis by assessing the effect of formic acid and fatty acids on invasion gene expression mediated solely through HilD. We constructed a Salmonella strain with null mutations in *hilC* and *rtsA* and placed *hilD* under a tetracycline-inducible promoter. Upon induction of *hilD* by tetracycline, *hilA* expression was induced, and this expression was reduced by fatty acids (Fig. S5). Preincubation with 10 mM formic acid, however, rescued this fatty acid-mediated repression, demonstrating the importance of *hilD* to this mechanism of regulation. To test the mechanism by which these signals control *hilD*, we analyzed the binding of fatty acids to the protein HilD with or without preincubation with formic acid using an enzyme-linked immunosorbent assay (ELISA). Fatty acids adherent to polystyrene plates were incubated with His-tagged HilD with or without prior preincubation with formic acid, and binding was detected using an anti-His antibody. As expected, HilD directly bound to c2-HDA (Fig. 3A). However, preincubation of HilD with formic acid significantly reduced its ability to bind to c2-HDA (dissociation constant [*K_d_*], 5.19 μM and 81.98 μM, respectively). We investigated this further by checking the binding of various classes of fatty acids having different carbon lengths and unsaturations. The *trans* isomer of c2-HDA with the same carbon length, t2-HDA, acted similarly, binding efficiently to HilD but poorly to HilD preincubated with formic acid (*K_d_*, 15.78 μM and 177.1 μM, respectively) (Fig. 3B). A longer *cis*-2 unsaturated fatty acid, c2-EA with 20 carbons, also bound to HilD but not to the formic acid preincubated protein (*K_d_*, 96.59 μM and 381.1 μM, respectively) (Fig. 3C). Oleic acid, a known intestinal fatty acid of 18 carbons and no *cis*-2 unsaturation, also showed a similar binding pattern (*K_d_*, 136.3 μM and 1373 μM, respectively) (Fig. 3D). Thus, formic acid preincubation prevented a wide range of fatty acids with different structures from interacting with HilD. This suggests that formic acid may competitively bind to a region of HilD that is required to bind to a chemical moiety common to all fatty acids.

10.1128/mbio.00012-23.5 FIG S5 *hilD* is essential for the function of formic acid as a derepressor. Expression of the invasion gene *hilA* was determined in Δ*hilC* Δ*rtsA tetRA-hilD*-*3×FLAG* Salmonella. Strains were left uninduced or were induced with 1 μg/mL tetracycline and preincubated with 10 mM formic acid followed by treatment with either 500 nM c2-HDA or an equal volume of DMSO. Invasion-gene expression is shown using a *hilA-luxCDABE* transcriptional reporter fusion. Luminescence was normalized to bacterial culture density. Data show the mean ± SD of five replicates. Download FIG S5, TIF file, 2.0 MB. Copyright © 2023 Chowdhury et al. 2023 Chowdhury et al. https://creativecommons.org/licenses/by/4.0/ This content is distributed under the terms of the Creative Commons Attribution 4.0 International license.

We have previously shown that specific amino acids of HilD are required to bind specific classes of fatty acids (15). Our current results show that formic acid preincubation prevented a range of fatty acids with various chemical structures from binding to HilD (Fig. 3A to D). We therefore tested whether formic acid could prevent the interaction of these repressive signals with HilD even though they utilize different protein binding moieties. We first tested a HilD^Q290A^ mutant, which can bind to fatty acids harboring a *cis*-2 unsaturation, such as c2-HDA and c2-EA, but fails to bind ligands such as oleic acid and t2-HDA that lack this structure (15). We found that HilD^Q290A^ directly bound to c2-HDA but failed to do so in the presence of formic acid (Fig. 3E). In contrast, the HilD^K293A^ mutant cannot bind fatty acids with a *cis*-2 unsaturation but does bind those that lack this structure. We found that HilD^K293A^ directly bound to oleic acid but lost that ability when the protein was preincubated with formic acid (Fig. 3F). These results conclusively show that formic acid competitively inhibits the interaction of HilD with all classes of fatty acids tested, to function as a derepressor.

### Formic acid prevents repressive fatty acids from disrupting the ability of HilD to bind DNA.

**FIG 4:**
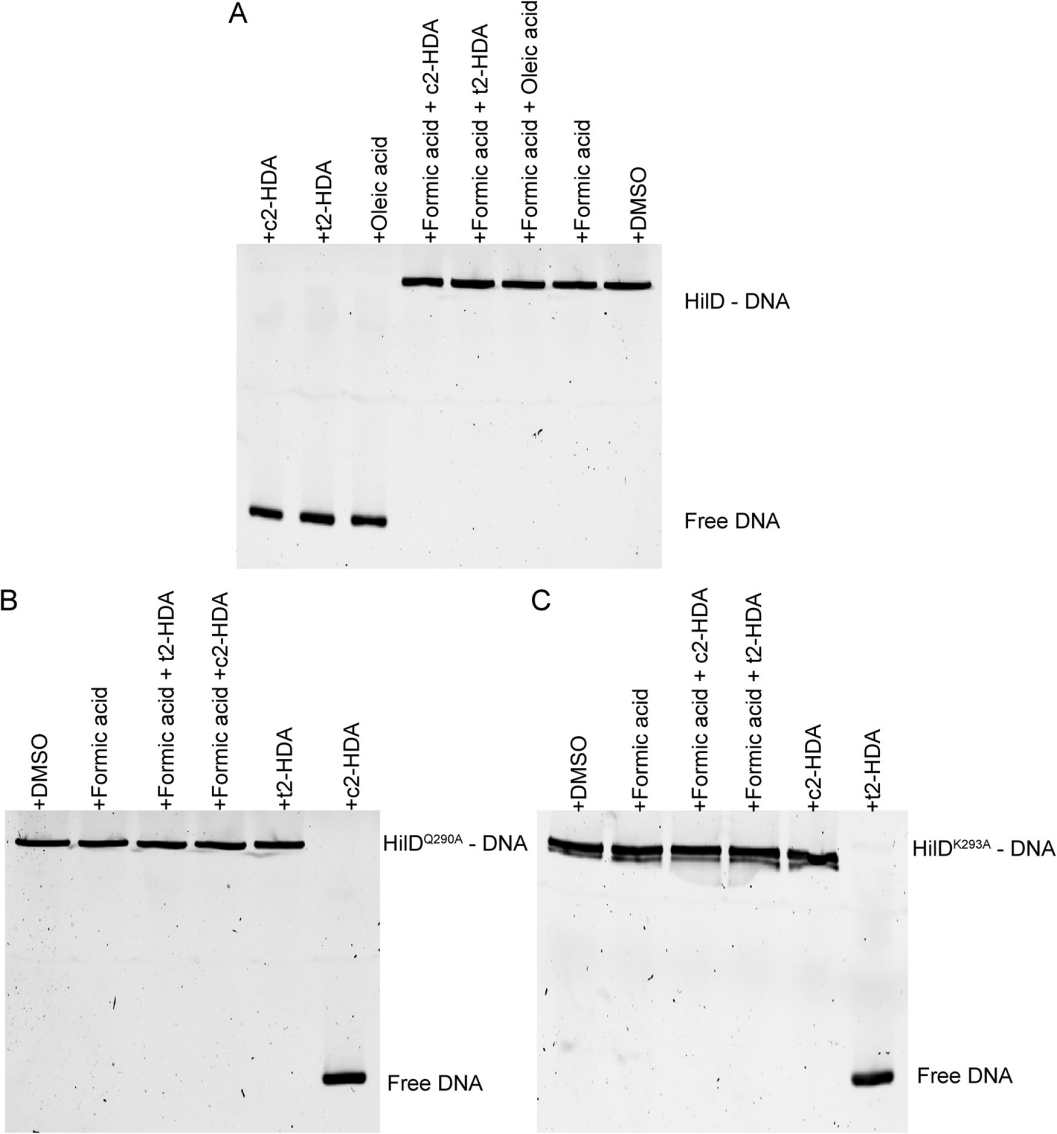
Formic acid prevents repressive fatty acids from disrupting the DNA-binding ability of HilD. (A to C) The effects of c2-HDA, t2-HDA, and oleic acid on the binding of (A) wild-type HilD, (B) HilD^Q290A^, or (C) HilD^K293A^ to *hilA* promoter DNA with or without preincubation with formic acid were assessed using EMSAs.

HilD binds in the *hilA* promoter region, initiating transcription and thus a cascade of invasion-gene expression, while repressive fatty acid signals prevent this binding (15). We reasoned that competitive binding by formic acid would thus allow the interaction of HilD with its DNA target even in the presence of these repressive signals. To test this, we performed an electrophoretic mobility shift assay (EMSA), assessing the effects of repressive fatty acids on the migration of a *hilA* DNA fragment in the presence of HilD with or without preincubation with formic acid. HilD bound to the *hilA* DNA, impeding its migration, while the fatty acids c2-HDA, t2-HDA, and oleic acid disrupted the HilD-*hilA* complex (Fig. 4A). However, preincubation of HilD with formic acid prevented this disruption, restoring the HilD-*hilA* complex. Additionally, formic acid prevented the disruption of the HilD^Q290A^-*hilA* complex by c2-HDA and that of the HilD^K293A^-*hilA* complex by t2-HDA (Fig. 4B and C). These results show that formic acid prevents all classes of fatty acids from disrupting the ability of HilD to bind to its target DNA. Together, they support a model by which Salmonella can utilize a microbial metabolite commonly found in the intestine to prevent a wide range of repressive signals from interacting with its principal invasion regulator.

### Formic acid preserves the stability of HilD in the presence of repressive fatty acids.

**FIG 5:**
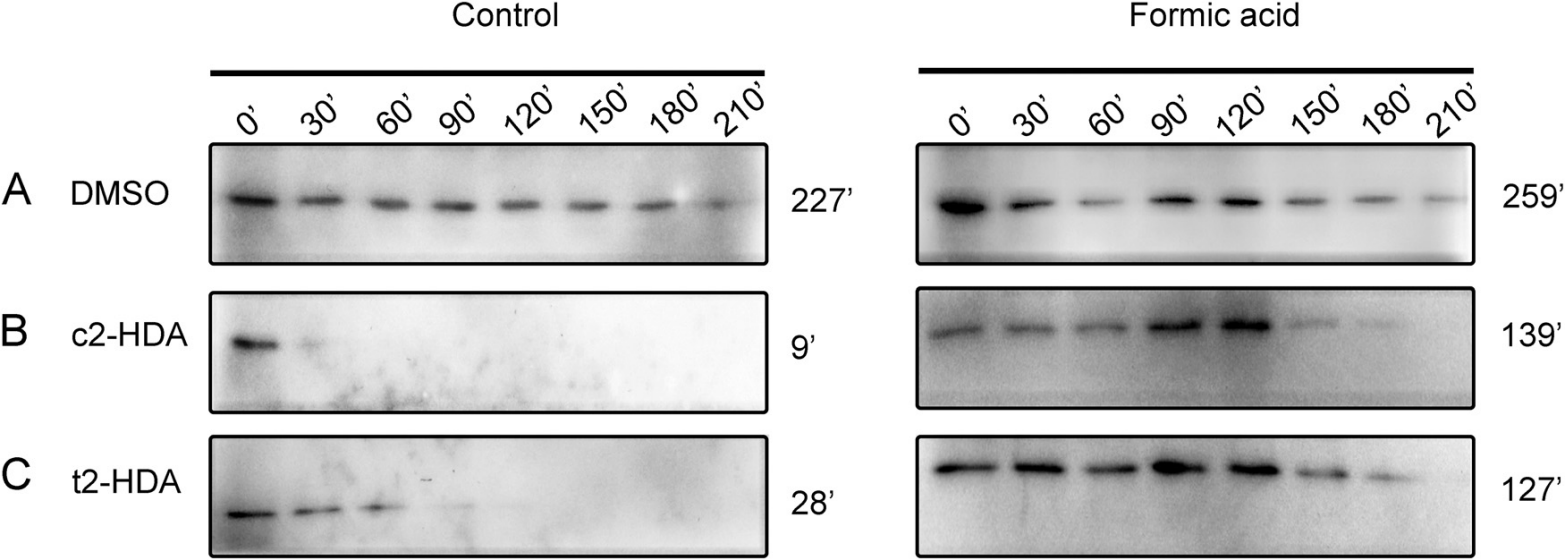
Formic acid preserves the stability of HilD in the presence of repressive fatty acids. (A to C) The stability of HilD in Salmonella carrying a chromosomal *tetRA-hilD-3×FLAG* tag, in the presence of either (A) DMSO, (B) c2-HDA 20 μM, (C) t2-HDA 20 μM, with or without preincubation with formic acid (10 mM) was evaluated by Western blot analysis. The half-lives after each treatment are indicated.

Growth of Salmonella in the presence of repressive fatty acids leads to a drastic reduction in the HilD half-life, which halts invasion gene expression (15, 22). As formic acid prevents fatty acids from binding to HilD, we reasoned that preincubation with formic acid may increase the stability of HilD in the presence of these fatty acids, thus sustaining expression of invasion genes. To test this hypothesis, we compared the stability of HilD in growing bacterial cultures, in the presence of fatty acids, with or without preincubation with formic acid. We used a Salmonella strain expressing *hilD* under the control of a tetracycline-inducible promoter and with a 3×FLAG tag that allows the quantification of HilD using Western blots. Salmonella cultures were grown with tetracycline to induce *hilD* expression, and equal numbers of bacteria were then preincubated with or without formic acid before the addition of repressive fatty acids. New protein production was stopped by using a cocktail of transcription- and translation-inhibiting antibiotics, and the stability of HilD was analyzed at subsequent time points. As shown in Fig. 5, treatment with c2-HDA led to rapid degradation of HilD, reducing its half-life from ~227 min to a mere ~9 min (Fig. 5B). Preincubation with formic acid, however, significantly increased the stability of HilD and extended its half-life from ~9 to ~139 min. Similar results were obtained with the stereoisomer t2-HDA, where formic acid preincubation increased the half-life of HilD significantly (from ~28 min to ~127 min) in the presence of t2-HDA (Fig. 5C). These results thus demonstrate a precise molecular mechanism by which Salmonella may exploit the ileal constituent formic acid to shield its primary invasion regulator HilD from interacting with repressive fatty acids, thus increasing its stability, and sustaining its DNA-binding ability in the presence of these compounds, thereby sustaining invasion gene expression.

### Salmonella utilizes formic acid in the murine ileum to induce invasion genes.

**FIG 6:**
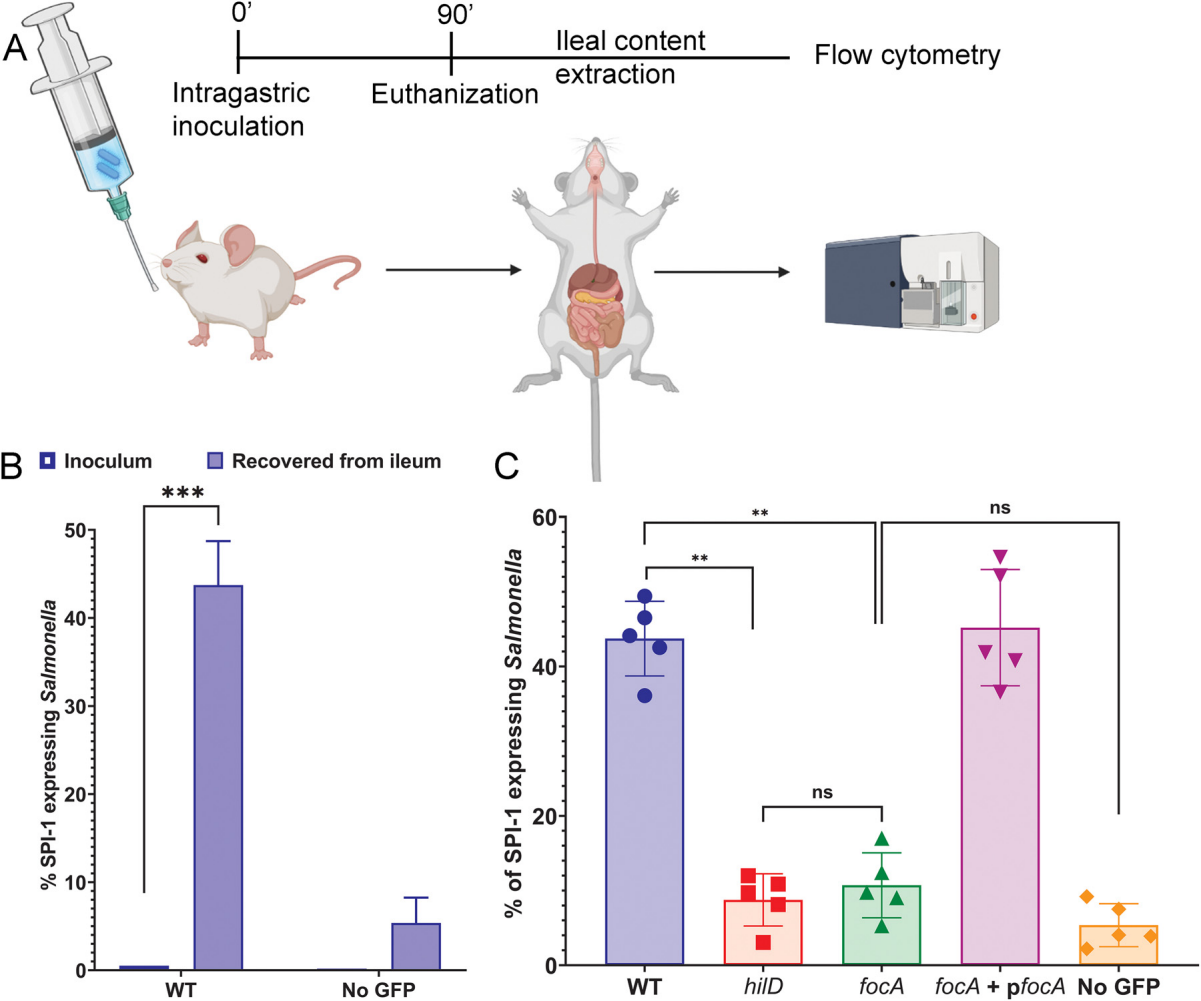
Salmonella utilizes formic acid in the murine ileum to induce invasion-genes. (A) Summary of the model used to analyze invasion gene expression in the murine ileum. (B) Salmonella strains constitutively expressing BFP were assessed for expression of the invasion gene *sicA* using a *sicA*-GFP reporter fusion in the inoculum and the ileal contents of C57BL/6 mice (*n* = 5), by flow cytometry. WT, wild type with P*sicA*-*GFP*; no GFP, a wild-type Salmonella strain without the GFP fusion. Bars represent the mean ± SD. The Mann-Whitney test was performed to calculate statistical significance. ***, *P* < 0.001. (C) Salmonella strains having different null mutations and constitutively expressing BFP were evaluated for expression of the invasion gene *sicA* using a *sicA-GFP* reporter fusion in the ileal contents of C57BL/6 mice (*n* = 5) by flow cytometry. Bars represent the mean ± SD. The Mann-Whitney test was performed to calculate statistical significance. **, *P* < 0.01; ns, not significant.

Our results show that Salmonella imports exogenous formic acid to sustain invasion-gene expression in the presence of repressive fatty acids. As the murine ileum is rich in formic acid, we hypothesized that Salmonella may utilize the formic acid present as a bona fide intestinal signal to derepress invasion in the ileum. To test this, we compared invasion gene expression in the murine ileum of a Salmonella wild-type strain with that of a *focA* mutant that is unable to import formic acid. To quantify gene expression, we used Salmonella strains with a green fluorescent protein (GFP) reporter fused to the promoter of the terminal invasion gene *sicA* (*PsicA→GFP*). Expression of invasion genes was prevented in cultures prior to animal inoculation, thus allowing us to assess their induction within the murine intestine. C57BL/6 mice were orally inoculated with equal numbers of bacteria, and after 90 min mice were euthanized, and bacteria were recovered from the ileum and analyzed by flow cytometry to evaluate *sicA* induction (Fig. 6A). We found that the proportion of the wild-type Salmonella population recovered from the ileum that demonstrated detectable *sicA* expression was ~44%, compared to ~0.5% in the inoculated culture (Fig. 6B), demonstrating clear induction of expression within the murine intestine. The Δ*focA* mutant, however, showed a significantly lower capacity to induce gene expression in the ileum, with *sicA* induced in only ~11% of its population (~4-fold less than the wild type) (Fig. 6C). In fact, invasion gene expression in the Δ*focA* mutant was not significantly different from that of a Δ*hilD* null mutant or of a control strain lacking the *PsicA→GFP* fusion, demonstrating complete repression due to the lack of *focA*. To ensure that these effects were indeed due solely to the loss of *focA*, we complemented the mutant using a plasmid constitutively expressing *focA*, finding that it restored *sicA* expression to the level of the wild type (~45%). These results show that Salmonella imports the formic acid present in the murine distal ileum and utilizes it as a signal to induce invasion genes in that region.

Short- and long-chain fatty acids that repress Salmonella invasion exist in high concentrations within the large intestine. We thus hypothesized that the lower concentration of formic acid present in this organ may not be sufficient to counter these repressive effects, thus providing a plausible mechanism of invasion induction. To test this hypothesis, we preincubated Salmonella with a lower concentration of formic acid (1 mM) prior to adding relatively higher concentration of fatty acids (40 μM c2-HDA) and assessed *sipC* gene expression. We found that formic acid preincubation was unable to rescue *sipC* repression caused by c2-HDA under these conditions (Fig. S6). As expected, both the *focA* mutant and the complemented strain remained repressed. These results show that the import of formic acid can prevent repression only when fatty acid concentrations are relatively lower in the surrounding environment.

10.1128/mbio.00012-23.6 FIG S6 Expression of invasion genes in the presence of low-formic acid and high-fatty acid concentrations. Expression of the invasion gene *sipC* was evaluated in Salmonella, preincubated with 1 mM formic acid prior to treatment with 40 μM c2-HDA or an equal volume of DMSO, using a *lacZY* transcriptional reporter fusion by β-galactosidase assay. Bars represent the mean ± SD (*n* = 3). Download FIG S6, TIF file, 1.3 MB. Copyright © 2023 Chowdhury et al. 2023 Chowdhury et al. https://creativecommons.org/licenses/by/4.0/ This content is distributed under the terms of the Creative Commons Attribution 4.0 International license.

## DISCUSSION

Bacterial pathogens have evolved to employ cues from their surroundings to optimize their virulence. Our data show that the enteric pathogen Salmonella uses competing signals within local environments to modulate its invasion of the intestinal epithelium, a necessary virulence function. Such regulation is essential because the expression of virulence genes is in most cases energetically costly and thus stringently controlled. As Salmonella navigates the small intestine, it encounters a high concentration of formic acid in the terminal ileum. This metabolite is then employed to competitively prevent repressive signals from binding to Salmonella’s principal invasion regulator, HilD, thus resulting in rapid induction of invasion. Fatty acids can directly bind to HilD, at least in part via their carboxylate moieties (15). We propose that formic acid, being a single-carbon carboxylate entity, can occupy the domain of HilD that is also required for the binding of intestinal fatty acid signals that potently inhibit the function of this invasion activator. Such binding would thus prevent other carboxyl group-containing compounds from accessing the putative HilD binding pocket. Indeed, our results show that preincubation with near-physiological concentrations of formic acid competitively prevents repressive fatty acids from binding to HilD and subsequently derepresses invasion gene expression. This model supports previous research (21) showing that Salmonella colonization preferentially occurs in the distal ileum, a region enriched in formic acid, and demonstrates an important mechanism by which a pathogen can utilize competition between signals in its local environment to accordingly regulate its virulence.

Our results suggest that formic acid may have much lower affinity for HilD than do fatty acids. Preincubation with formic acid at a high concentration (at the millimolar level) prevented invasion gene repression by fatty acids. In contrast, when Salmonella was preincubated with low concentrations of fatty acids (at micromolar levels) invasion gene expression remained low and could not be rescued by the addition of formic acid (Fig. 1F and Fig. S2). If formic acid had higher affinity for HilD than to the fatty acids, it might replace the fatty acids from HilD and thus rescue the invasion gene expression. These data indicate that, despite its lower affinity, formic acid can outcompete the fatty acid signals and sustain Salmonella invasion when the pathogen is exposed to formic acid prior to the fatty acids and in much higher concentrations. In addition, these data also suggest that formic acid competitively inhibits fatty acids from binding to HilD. If formic acid were an allosteric or a noncompetitive inhibitor, increasing the concentration of fatty acids would not overcome the derepression by formic acid. Therefore, adding higher concentrations of fatty acids to formic acid-preincubated HilD would likely result in sustained activation of HilD and thus high invasion gene expression, but we found that adding higher concentrations of fatty acids can overcome the derepression by formic acid, and thus invasion genes are repressed (Fig. S7). This suggests that formic acid may act as a competitive inhibitor.

10.1128/mbio.00012-23.7 FIG S7 Expression of invasion genes in the presence of a high concentration of fatty acid. Expression of the invasion gene *hilA* was determined in Salmonella with different treatments (preincubation with 10 mM formic acid followed by either 20 μM c2-HDA or 80 μM oleic acid or an equal volume of DMSO) shown using a *hilA-luxCDABE* transcriptional reporter fusion. Luminescence was normalized to bacterial culture density. Data show the mean ± SD of five replicates. Download FIG S7, TIF file, 1.9 MB. Copyright © 2023 Chowdhury et al. 2023 Chowdhury et al. https://creativecommons.org/licenses/by/4.0/ This content is distributed under the terms of the Creative Commons Attribution 4.0 International license.

As the expression of the virulence machinery is expensive, enteric pathogens stage their expression at appropriate locations by perceiving the relative concentrations of certain compounds in the adjacent milieu. Relatively higher concentrations of these compounds can shift the balance from survival and proliferation toward expression of virulence genes. Our model suggests that, as Salmonella encounters the high proportion of formic acid in the terminal ileum, this small molecule is used as a spatial cue that shifts the balance toward invasion in the terminal ileum. In contrast, Salmonella encounters an environment rich in repressive fatty acids and low in formic acid (1, 14, 26, 27) in the large intestine, which can be utilized as a spatial cue to shift the balance toward proliferation. This reduces the costly expression of invasion determinants and conserves energy for proliferation. Thus, Salmonella has adapted to utilize these spatial cues to acutely sense the appropriate location for its invasion.

There are additional important benefits to Salmonella in inducing invasion in the upper intestine. The inflammation that accompanies invasion changes the luminal environment in a way that favors the growth of the pathogen. Indeed, studies have shown that in such inflammatory environments, the ability of Salmonella to utilize formic acid for respiration and oxidation provide a crucial survival advantage (28). As Salmonella carries formate dehydrogenase (Fig. 2), it can survive and multiply in such situations. Thus, Salmonella benefits by using formic acid in two ways, to derepress invasion and to survive and proliferate in the inflamed intestinal environment.

The advantages of such a regulatory system for Salmonella are clear. Yet this species is but one of several pathogens that might avail themselves of similar cues and mechanisms to control their virulence. Fatty acids are known to be crucial modulators of virulence in enterohemorrhagic Escherichia coli (EHEC) (14, 29, 30), Vibrio cholerae (31, 32), and Listeria monocytogenes (33–35). In most cases, fatty acids repress virulence genes in these organisms by directly binding to a virulence master transcriptional regulator, disrupting its ability to bind DNA. As the chemical cues we describe here are common intestinal constituents, it is reasonable to speculate that a similar mechanism of chemical repression and derepression of virulence genes may exist in other important enteric pathogens. Further research will be required to determine whether and how the interplay of competing intestinal signals more broadly modulates the virulence of intestinal pathogens.

## MATERIALS AND METHODS

### Bacterial strains.

All experiments were performed using Salmonella enterica subsp. *enterica* serovar Typhimurium 14028s or E. coli BL21 DE3 unless otherwise noted. All strains, plasmids and primers used in the study are listed in Table 1 and Table 2 respectively. Fatty acids c2-EA (93772-82-8), oleic acid (90260), and t2-HDA (11132) were purchased from Cayman Chemicals. c2-HDA was purchased from both Cayman Chemicals (11133) and Larodan (10-1609). Propionic acid was purchased from Sigma (W292400). All antibiotics were used at their standard concentrations (chloramphenicol, 25 μg/mL; kanamycin, 50 μg/mL; ampicillin, 100 μg/mL) unless otherwise mentioned.

**TABLE 1:** Strains used in the study

Strains and plasmids	Description	Source or reference
Strain no.		
CA32	Wild-type 14028s	American Type Culture Collection
CA5399	p*hilA-luxCDABE*	This study
CA5389	Δ*hilC* Δ*rtsA*::*kan tetRA-hilD-3XFLAG* p*hilA-luxCDABE*	This study
CA412	*sipC*::*lacZY*	40
CA5254	Δ*focA*::*kan* p*hilA-luxCDABE*	This study
CA5398	Δ*pflB*::*kan* p*hilA-luxCDABE*	This study
CA5403	Δ*focA* Δ*pflB*::*kan* p*hilA-luxCDABE*	This study
CA5239	*sipC*::*lacZY* Δ*fdoG* Δ*fdnG* Δ*fdhF* p*hilA-luxCDABE*	This study
CA5462	Δ*focA*::*kan sipC*::*lacZY* p*CA280*	This study
CA5464	Δ*focA*::*kan sipC*::*lacZY* p*BR322*	This study
CA5046	E. coli BL21 DE3 pET15b-*hilD*	15
CA5055	E. coli BL21 DE3 pET15b-*hilD^Q^*^290A^	15
CA5056	E. coli BL21 DE3 pET15b-*hilD* ^K293A^	15
CA2446	*tetRA-hilD-3XFLAG attλ:pDX1*::*hilA'-lacZ*	7
CA3887	Δ*phoN*::*BFP*, P*sicA*→*GFP*	40
CA4178	Δ*hilD*::*kan ΔphoN*::*BFP*, P*sicA*→*GFP*	40
CA5287	Δ*focA*::*kan ΔphoN*::*BFP*, P*sicA*→*GFP*	This study
CA5474	Δ*focA*::*kan ΔphoN*::*BFP*, P*sicA*→*GFP* pCA280	This study
CA3730	Δ*phoN*::*BFP*	This study
Plasmid		
pBA426	*hilA-luxCDABE*	16
pCA280	pBR322-*focA*	This study
6XHis-hilD	*hilD*-pet15b	15
6XHis-hilD^Q290A^	*hilD*^Q290A^-pet15b	15
6XHis-hilD^K293A^	*hilD*^K293A^-pet15b	15

**TABLE 2:** Primers used in the study

Name	Sequence
FOCADWFP	GTTAGTATCTCGTCGCCGACTTAATAAAGAGAGAGTTAGTGATTGTGTAGGCTGGAGCTGCTTCGAAGTTCCTAT
FOCADWRP	GTGGATTTTTTATTTACTGCGTGTAATGGGCATCAACAGACCATGGTCCATATGAATATCCTCCTTAGTTC
FOCA-PBR322FP	ATCGCTAGGGATCCACTTTAAGAAGGAGATATACCAGTGAAAGCTGACAACCCTTTTGAC
FOCA-PBR322RP	TCGATGACGTCGACTTAATGATGCTCGTTACCACGCAGG

### Construction of Salmonella mutant strains.

Salmonella strains with deletions in various genes were constructed as previously described (16, 22). Using plasmid pKD4, the DNA region encoding resistance against kanamycin was amplified by PCR with a 40-bp homology extension flanking *focA*. The PCR fragment was electroporated into a strain expressing λ Red recombinase (36). Loss of *focA* was confirmed using PCR and sequencing. Mutations were unmarked by the pCP20 recombinase protocol (37). P22 transduction was used to transfer constructs among strains (38).

### Luciferase assay.

Salmonella strains carrying *luxCDABE* reporter plasmids were grown overnight in 5 mL LB broth with appropriate antibiotics. These were subcultured (1:100) in M9 minimal medium with 0.2% glucose and antibiotics for 16 h. Equal numbers of bacteria (optical density at 600 nm [OD_600_] of 0.02) were inoculated into LB broth containing 100 mM MOPS (morpholinepropanesulfonic acid), pH 6.7, and antibiotics with 10 mM formic acid or left untreated. These were incubated for 3 h at 37°C in an incubator without aeration. Dimethyl sulfoxide (DMSO) or different concentrations of fatty acids were added to the reaction, and replicate aliquots were introduced into the BioTek Synergy H1 microplate reader. Luminescence and OD_600_ were recorded every half-hour for 24 h. Data were analyzed in GraphPad Prism. For every treatment, the area under the curve (AUC) was calculated. DMSO was normalized to 100%, and other reactions were calculated accordingly.

### β-Galactosidase assay.

Salmonella strains carrying *lacZY* fusions were preincubated with 1 or 10 mM formic acid for 3 h and grown in 1 mL LB broth with 100 mM MOPS, pH 6.7, antibiotics, and different concentrations of fatty acids or an equal volume of DMSO, without aeration for 16 to 18 h. The β-galactosidase assay was performed using the method of Miller, as previously described in detail (39). Miller units were calculated using the following equation: Miller units = (OD_420_ × 1,000)/(OD_600_ × *t* × *v*), where *t* is the time (minutes) the reaction was allowed to continue before adding the stop solution, *v* is the volume of culture (mL) assayed, and OD_420_ and OD_600_ are the densities of the cultures at 420 and 600 nm, respectively. All reactions were performed in quadruplicates. Data were analyzed in Excel and plotted in GraphPad Prism.

### Measurement of formic acid concentration.

Mice were dissected, and distal ileum (~6 cm above the ileocecal junction), whole cecum, and colon (~6 cm from the end of cecum) were collected. The contents were extracted in 1 mL of PBS, vortexed vigorously, and centrifuged at 5000 × *g* for 10 min to precipitate debris. Supernatants were collected for measuring formic acid concentration. These were measured using an enzymatic bioanalysis detection kit, following the manufacturer’s protocol (R-Biopharm). This method uses the enzymatic conversion of formic acid and NAD^+^ to bicarbonate and NADH through the action of the enzyme formate dehydrogenase. The NADH concentration is then measured at 340 nm to calculate the formic acid concentration. A standard curve was plotted before the experiment to correlate concentrations and optical densities.

### ELISA.

Assays were performed as previously described in detail in Chowdhury et al. (15, 16). Briefly, 96-well polystyrene plates coated with various concentrations of fatty acids in coating buffer (0.05 M sodium carbonate-bicarbonate, pH 9.3) were incubated overnight at 4°C. The wells were washed three times with PBS, followed by the addition of 200 μL blocking buffer (1% Ficoll 400 in PBS). After 2 h of blocking at room temperature, the wells were washed three times with PBS, and different concentrations of proteins, preincubated with 500 μM formic acid, in blocking buffer were added and incubated at room temperature for 2 h. The wells were washed, incubated with a 1:1,000 dilution of anti-His tag antibody, and incubated at room temperature for an hour. After washing, the wells were incubated with a 1:5,000 dilution of anti-mouse secondary antibody for an hour. Finally, the wells were washed three times with PBS-T (PBS plus 0.1% Tween 20), and 100 μL OPD (o-phenylenediamine) substrate was added. After color development, the reaction was ended with 1 N sulfuric acid and read spectrophotometrically at 490 nm. Data were analyzed in GraphPad Prism. Nonlinear regression was plotted, and one-site total binding was performed to calculate *K_d_* values.

### EMSA.

Assays were performed as previously described (15, 16). HilD, HilD^Q290A^, or HilD^K293A^ (200 μM) was preincubated with either 500 μM formic acid or an equal volume of PBS for 1 h. *hilA* promoter DNA (–286 to +31) (50 nM) was added to the reaction in binding buffer (100 mM Tris, pH 7.5, 10 mM EDTA, 1 M KCl, 1 mM dithiothreitol [DTT], 50% vol/vol glycerol). Various concentrations of the fatty acids (c2-HDA, 20 μM; t2-HDA, 40 μM; or oleic acid, 50 μM) or control solvents were added, and the reaction was incubated at room temperature for 1 h. Dye solution (10 mM Tris, 1 mM EDTA, 50% vol/vol glycerol, 0.001% wt/vol bromophenol blue) was added to a final concentration of 1×, and samples were separated on 7% acrylamide gels. DNA was stained using SYBR Safe and visualized in a Bio-Rad GelDoc imaging system.

### HilD degradation assay.

Assays were performed as previously described (22). Salmonella strains carrying a chromosomal tetracycline-inducible *hilD* with a C-terminal 3×FLAG tag and a *hilA*::*lacZY* fusion (for invasion gene expression assessment) were grown overnight in LB and then diluted 1:100 into LB buffered with 100 mM MOPS (pH 6.7) with 2.5 μg/mL tetracycline. This concentration of tetracycline induced *hilD* to wild-type levels. After 3 h of growth, cultures were treated with 10 mM formic acid or left untreated and incubated at room temperature for 3 h without aeration, followed by fatty-acid treatment. After 1 h of incubation at 37°C with aeration, samples were collected to measure *hilA*::*lacZY* expression in a β-galactosidase assay. Individual cultures were then equilibrated to an OD_600_ of 1. New protein production was halted by adding an antibiotic combination (100 μg/mL rifampin, 200 μg/mL streptomycin, and 50 μg/mL spectinomycin). These cultures were incubated at 37°C with aeration, and 200-μL aliquots were collected every 30 min for 4 h. Samples were lysed in 5× Laemmli buffer, boiled, and immunoblotted with an anti-FLAG antibody to monitor HilD levels.

### HilD protein expression.

*hilD* alleles were cloned into pET15b(+), expressed, and purified as described in detail previously (15). Briefly, the pET15b-6XHis-hilD or pET15b-6XHis-hilD^Q290A^ or pET15b-6XHis-hilD^K293A^ plasmid was transformed into E. coli BL21(DE3) for expression. Transformants were induced with 5 mM IPTG (isopropyl β-d-1-thiogalactopyranoside) for 4 h. Cultures were centrifuged, lysed, treated with TALON beads, and applied to protein purification columns. Bound protein was eluted and confirmed by SDS-PAGE. Following confirmation, eluted samples were dialyzed overnight. The final protein concentration and quality after dialysis were assessed by Bradford estimation and SDS-PAGE.

### Ethics statement.

Studies involving vertebrate animals were approved by the Cornell University Institutional Animal Care and Use Committee (protocol 2012-0074). Euthanasia was performed using carbon dioxide inhalation in accordance with the American Veterinary Medical Association Guidelines for Euthanasia of Animals. The Cornell University Animal Care and Use program and associated animal facilities are operated in accordance with the U.S. Department of Agriculture Animal Welfare Act (1966), Regulation (C.F.R., 2009), and policies, the Health Research Extension Act (1985), the Public Health Service Policy on Humane Care and Use of Laboratory Animals (PHS, 2002), the Guide for the Care and Use of Laboratory Animals (NRC, 2011), the Guide for the Care and Use of Agricultural Animals in Research and Teaching (2010), the New York State Health Law (Article 5, Section 504), and other applicable federal, state, and local laws, regulations, policies, and guidelines.

### Mouse experiments.

Salmonella strains, either wild type or with different null mutations, carrying a constitutive blue fluorescent protein (BFP) only (*phoN*::*BFP*, as negative control) or with a GFP reporter fusion to the promoter of invasion gene *sicA* (P*sicA*→*GFP*), were grown overnight in LB broth with 1 mM nonanoic acid (to stop the expression of invasion genes). On the day of the experiment, they were subcultured 1:100 in LB broth with 1 mM nonanoic acid and grown to an OD_600_ of 1. Equal volumes of cultures were precipitated, washed twice, and resuspended in 1 mL PBS. Female C57BL/6 mice (*n* = 5), 6 to 8 weeks of age, were gavaged with 100 μL of 3% sodium bicarbonate prior to bacterial inoculation (4 × 10^8^ CFU in 100 μL PBS). As a control, 100 μL of the inoculum was stored on ice. Feed was removed 2 h before inoculation and supplied again after infection. After 90 min of infection, mice were euthanized, and the distal ileum (~6-cm length above the cecum) was extracted. All contents were transferred to 1 mL chilled PBS. Samples were filtered through a 5-μm filter to eliminate debris and centrifuged to precipitate bacteria. Inoculum was precipitated similarly, and all bacterial pellets were fixed in 1 mL of 4% PFA (paraformaldehyde) in PBS, on ice and shielded from direct light, for 30 min. Following fixation, the fixative was aspirated, and the pellet was resuspended in 1 mL PBS. These samples were analyzed by flow cytometry (Thermo Fisher Attune NxT analyzer), gating on the BFP-expressing population (to determine total Salmonella counts), and calculating the proportion of Salmonella expressing invasion genes (expression of both BFP and GFP). Data were plotted in GraphPad Prism. Illustrations were created in BioRender.

### Statistical analysis.

All experiments were repeated at least twice. Statistical significances were calculated in GraphPad Prism using the Mann-Whitney test. Differences were considered significant when the *P* value was <0.05, <0.01, or <0.001, indicated by *, **, and ***, respectively.

### Data availability.

All relevant data have been provided in the manuscript and the supplemental information files.
