# Supplementary figures and images for: Salmonella Invasion Is Controlled by Competition among Intestinal Chemical Signals
Source: mBio. 2023 Apr 5;14(2):e00012-23. doi: 10.1128/mbio.00012-23 (PMC10127606; doi:10.1128/mbio.00012-23)

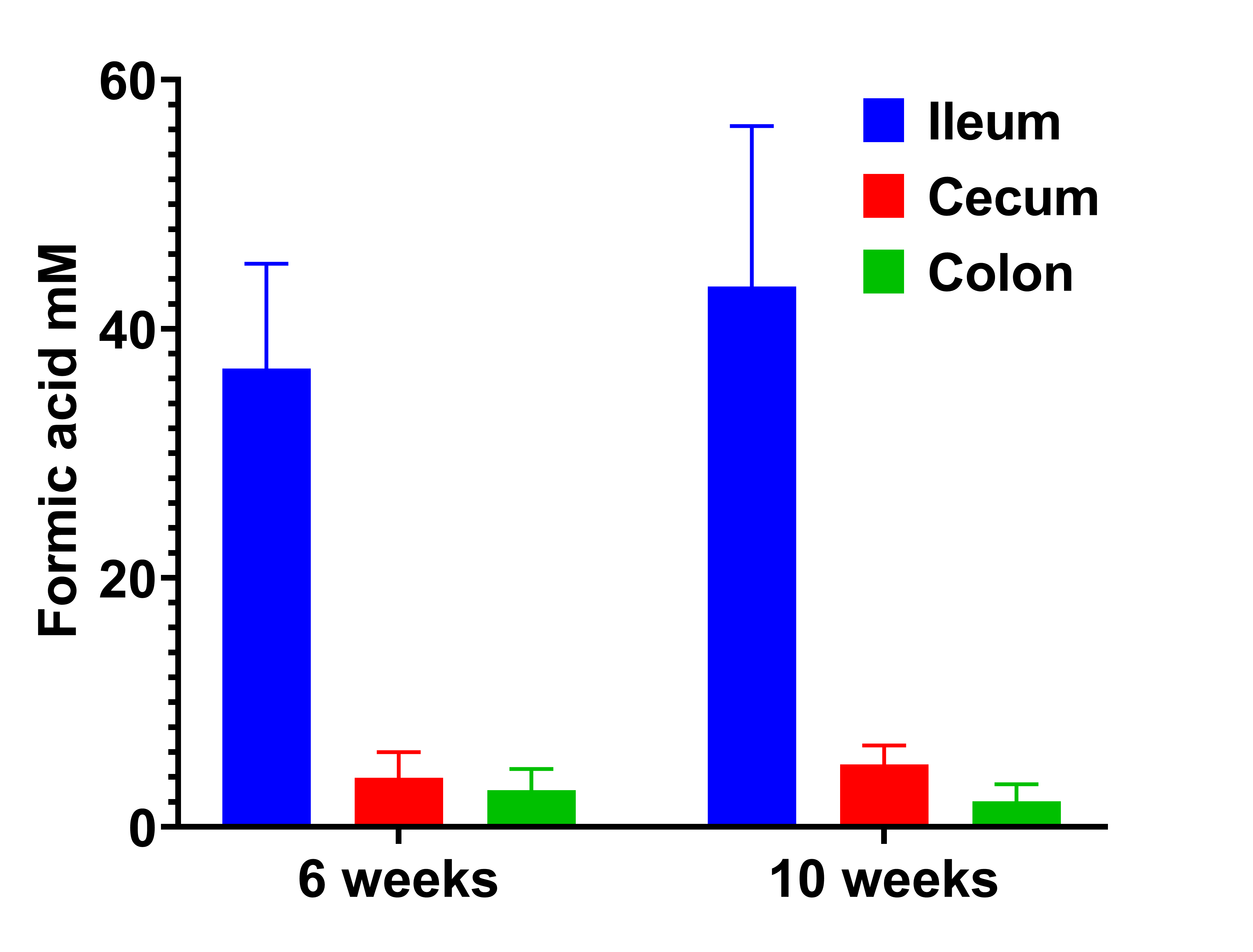

Supplement: FIG S1 [file mbio.00012-23-s0001.tif]

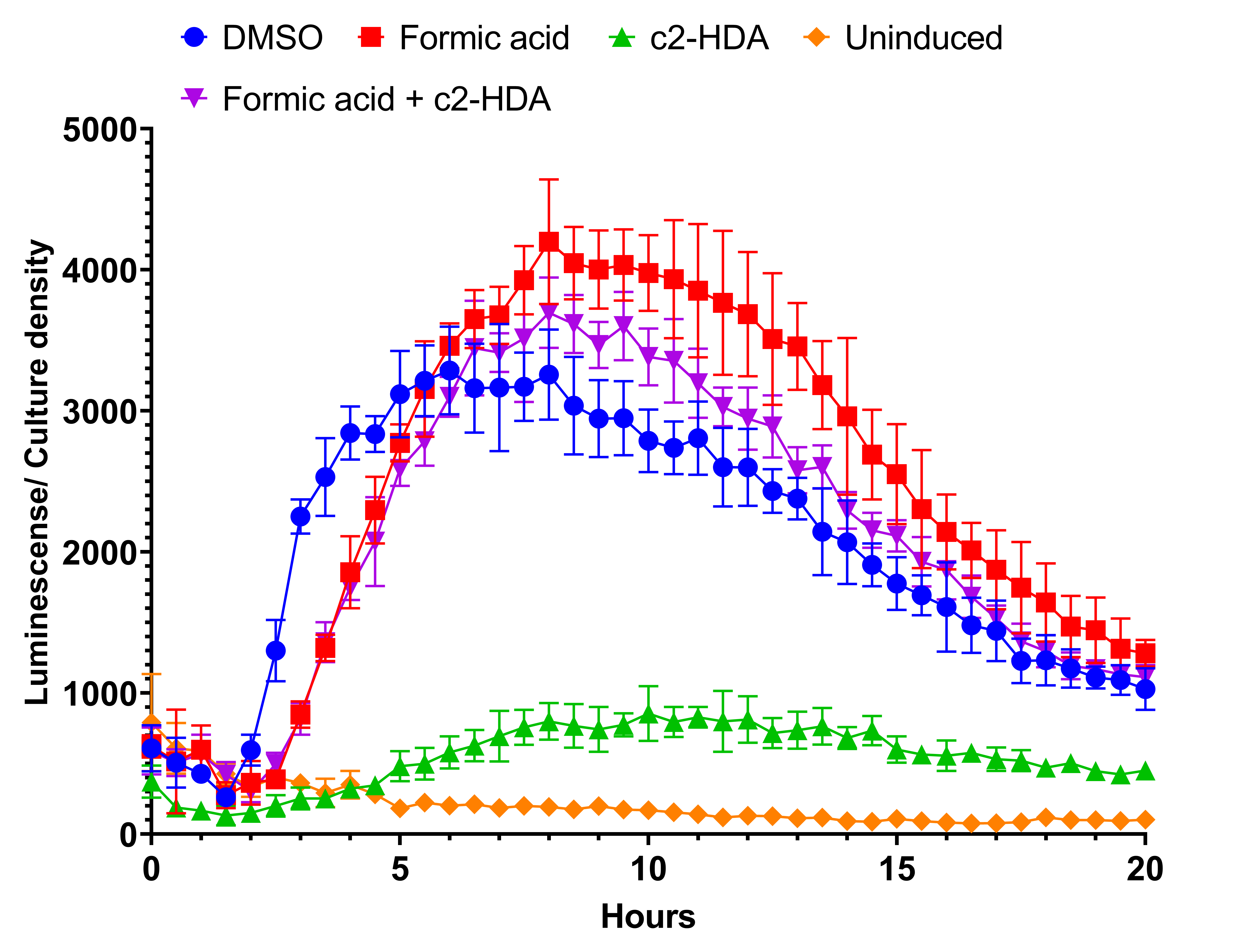

Supplement: FIG S5 [file mbio.00012-23-s0005.tif]

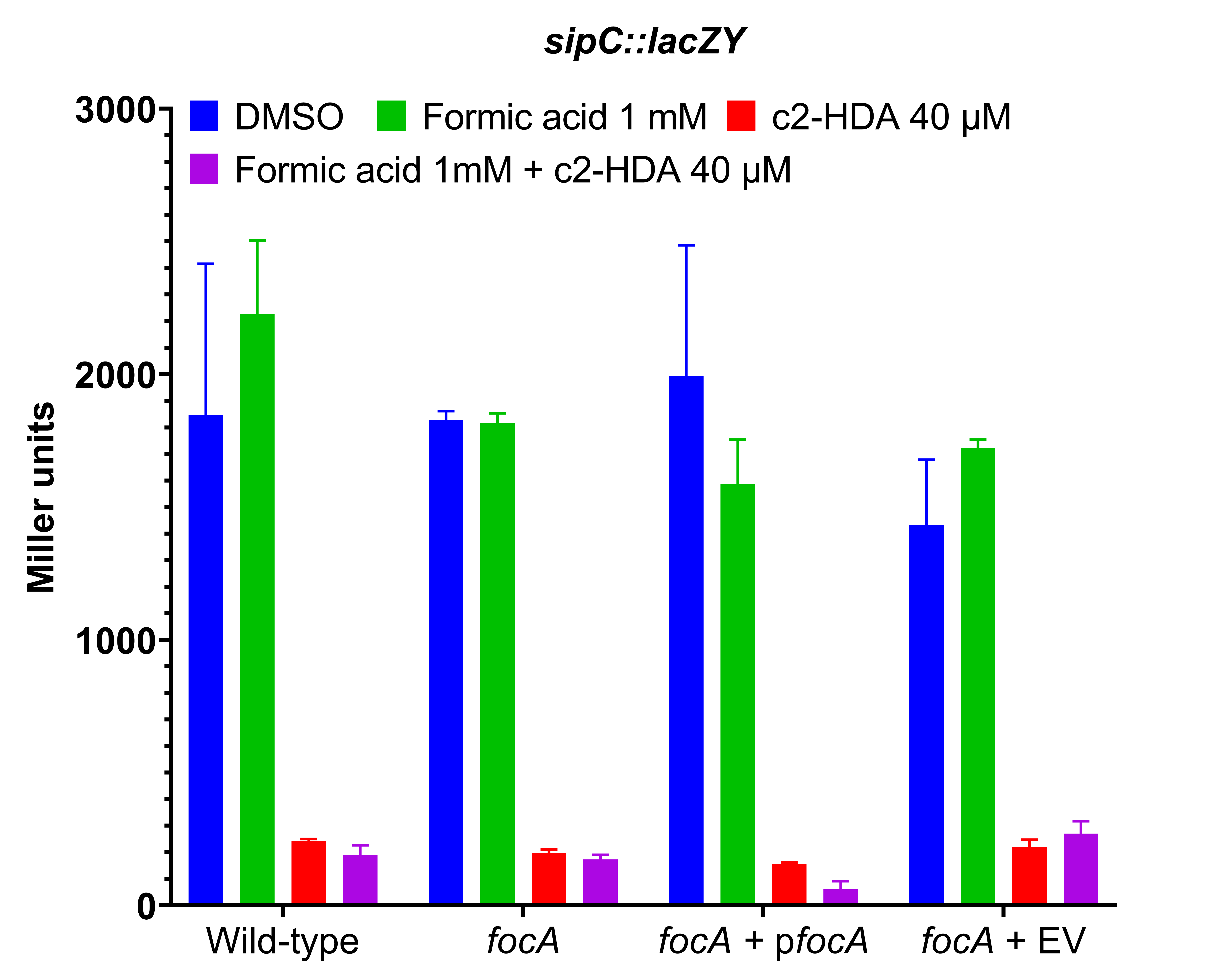

Supplement: FIG S6 [file mbio.00012-23-s0006.tif]
